# Homage to James Feeney, a pioneer of biological NMR – Part I

**DOI:** 10.3389/fmolb.2026.1758506

**Published:** 2026-03-05

**Authors:** Andrew Lane

**Affiliations:** University of Kentucky, Lexington, KY, United States

**Keywords:** DHFR, Enzymes, large-scale facilities, NMR, structural biology

Jim Feeney (1936-2025) (Figure 1) was a prominent figure in the field of biological nuclear magnetic resonance (NMR), who spent most of his career at the National Institute for Medical Research in Mill Hill, London. With his scientific expertise and personality, he positively impacted the professional and personal lives of many people who worked with him. I worked with him from 1986 until 2001. Jim had a long and productive career from the early days of applying NMR to chemical and biochemical problems. He had a firm grasp of NMR, often quite intuitively. He showed me how to interpret scalar spin couplings, how to think about chemical exchange, and the importance of NMR in functional analysis of macromolecules. In addition to his many achievements in molecular pharmacology, as a writer and editor, and as a leader, he was also an exceptionally kind person who always made time for. He was instrumental in hiring me to NIMR in my first academic position, and was steadfastly supportive both of me and to my wife Teresa Fan who visited us every year. Jim was a terrific boss, a true mentor, and a very dear friend. He will be sorely missed in so many ways, but his legacy will live on.

**FIGURE 1:**
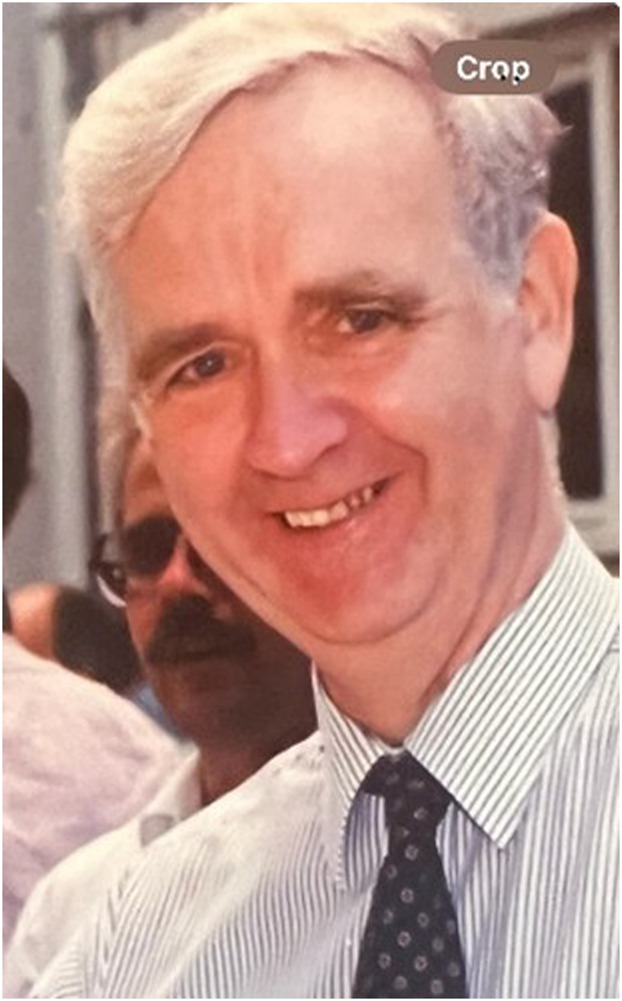
A photo of Jim at NIMR in the late 90s.

In these two joint tributes to Jim’s work, we collected the testimony of colleagues/friends that focus on how his scientific approach benefitted the NMR comminity, his warmth and humanity and display the breadth of his impact. A detailed scientific obituary is published in the review journal that Jim co-founded and edited^1^. A short obituary has also appeared in the website of International Society of Magnetic Resonance (ISMAR)^2^.

## Berry and Nigel Birdsall, NIMR, London United Kingdom

We met Jim in 1970 and did my PhD with Jim at Cambridge University, Pharmacology Department. Berry worked directly with him at NIMR 1972 to 2011 and was the luckiest person, to have done her PhD with Jim and then worked closely with him for 40 years followed by 15 more years of friendship. Working with Jim was always fun. Jim created an environment of cooperation where it was fun to do research. When an experiment went particularly well or there was an exciting new result, he would stand up, run across the room and bowl an imaginary cricket ball. He loved singing at conferences and wrote many versions of the song ‘Much Binding in the Lab’ for important lab events. It was always a joy to work with Jim.

## Paul Driscoll, the crick institute, London United Kingdom

I was Group Leader at NIMR from 2008 to 2014. I primarily remember Jim as a ‘fixture’ on the NMR conference circuit during my earlier years in the field, and as the Director of the MRC Biomedical NMR Centre at NIMR-Mill Hill at which for many years my group at UCL benefited as an external user. Eventually I migrated to a Group Leader position at NIMR and enjoyed more directly, as an internal user, the excellent facilities that Jim had brought together for the study of biological macromolecules by NMR. Whether Jim was directly involved in my appointment is unclear to me, but he was likely an influence in the background and for that I will always be grateful. I would like to recall specifically what is to me the classic work on ligand complexes of DHFR and, in particular, the observation of correlated motions involving arginine side chain NH_2_ groups. It was this work that allowed us and others to rationalise similar behaviour in complexes of SH2 domains with phosphotyrosine peptides. Regarding the NMR Centre I often heard its operations spoken of as the ‘right way’ to run a national facility. The credit for that description must sit with Jim, and his choice of staff, together with his gentlemanly dealings with peers and juniors alike. Jim was undoubtedly one on the nicest people in the field, and I am glad to have known him.

## Tom Frenkiel, the crick institute, London United Kingdom

I was at NIMR from 1983 until it became part of the Crick in 2015, although Jim of course retired well before then. Jim was a remarkable person in many ways. I consider myself privileged to have worked under his direction at the MRC Biomedical NMR Centre at Mill Hill, which I did for almost half of my working life. The Centre started operation in January 1981, at which point it was equipped with a 200 MHz spectrometer. I strongly suspect that Jim was the driving force behind the creation of the Centre; he was certainly its first director, though the title he had was Centre Controller, which he intensely disliked but had been thought necessary to avoid the confusion that could have occurred had there been a Director of NMR as well as the Director of NIMR. Title aside, Jim set the direction and, in some ways more importantly, the ethos of the Centre: from the outset its user community included both extramural and intramural research groups, and one of Jim’s many insights was to see that to be successful the Centre should serve the needs of both of these groups of users equally well. And the Centre most definitely was successful under Jim’s leadership: a 500 MHz instrument was added within a few years, followed by additional instruments at ever higher field strengths, ensuring that users of the Centre during this period had access to the highest field strengths available in the United Kingdom.

A second factor behind the Centre’s success and relevance was Jim’s skill in identifying the novel NMR methods and emerging technical developments that would be advantageous to users of the Centre. One example is three-dimensional NMR; Chris Bauer and I were given every encouragement to implement this at the Centre well before the capability was available commercially. I also remember that Jim was very quick to see the potential of cryogenically cooled probes and pulsed-field gradients; in both cases I initially saw drawbacks and disadvantages but Jim saw benefits and advantages.

At a personal level, I cannot imagine there being a better person to work for than Jim. He gave me lots of freedom and certainly did not micro-manage; working for Jim seemed more like a collaboration than an employee-manager relationship. He brought a wry, almost impish, sense of humour to the workplace and he was hugely supportive of the people for whom he was responsible.

Above all, Jim was an excellent role model and mentor. I particularly admired, and attempted to emulate, the effectiveness with which Jim operated in meetings such as those of the NMR Centre’s Advisory Committee; he was never confrontational, but relied instead on carefully thought-out arguments to achieve his desired outcomes. I think this is what made Jim the remarkable person that he was: thoughtful, thoroughly decent, considerate, respectful of others, wise beyond measure. He will be sorely missed.

## Tony Holder, the crick institute, London United Kingdom

I was at NIMR from 1988 to 2015. Malaria parasites cause disease by invading red blood cells, and antibodies binding to their surface stop them from doing so. A very productive collaboration with Jim and his team solved the structure of the target of these antibodies and resolved the specificity of the binding. This tremendous achievement was a first in the malaria world and shaped how we thought about developing a malaria vaccine. Twenty-five years later his early work continues to shape current research. Jim was characteristically modest about his contribution and yet its impact was profound.

## Geoff Kelly, the crick institute, London United Kingdom

I joined the MRC Biomedical NMR Centre at NIMR in 1999, just a couple of years before Jim’s official retirement (he of course remained active in science for many more years, most notably as co-editor of *Progress in Nuclear Magnetic Resonance Spectroscopy*, a journal he had co-founded). Jim’s remarkably broad, deep and intuitive understanding of NMR and enzymology was immediately apparent; this, coupled with his approachability, made him the point of reference for anyone with unusual results to interpret or observations to rationalise.

Perhaps even more apparent was the deep affection he inspired in his students and co-workers. A true gentleman, Jim will be remembered with huge fondness by generations of colleagues, for his meticulous approach to scientific enquiry, his insight and incisiveness, his quiet wisdom and sound guidance, but perhaps most of all for his kindness, gentleness and patience.

## Vladimir Polshakov, Lomonosov Moscow state university, Moscow, Russia

I first met Jim Feeney in 1989 when I came to work as a postdoc in his lab at Mill Hill with a 4-month placement in the Molecular Structure Division, which concluded in February 1990. I then worked with him again for 2 years, from 1992 to 1994. Starting in 1995, we began a close, long-distance collaboration between London and Moscow, supported by the Howard Hughes Medical Institute and the Wellcome Trust, which continued for 10 years until his retirement. Throughout this decade, I typically spent one to 3 months/year working at Mill Hill. Although Jim formally retired in the early 2000s, he continued active scientific work for many years afterwards. Our last joint paper, published in 2010, was also his final scientific publication, a fitting conclusion to his remarkable career. I am proud to call myself one of Jim Feeney’s students and postdocs. He taught me so much about protein–ligand interactions, structural biology, biochemistry, and, of course, NMR. I will never forget his lessons on writing papers and grant proposals, or his quiet but precise way of guiding ideas to clarity. Jim had also a wonderful sense of humor, something everyone enjoyed during his after-dinner talks at the close of conferences. Beyond science, he was a warm and generous friend.

## Andres Ramos, university college London, London, United Kingdom

During my years as a postdoctoral fellow in the Molecular Structure Division of the MRC National Institute for Medical Research, Jim Feeney represented a powerful example of how innovative and world-class science is done as a part of a community of researchers and academics. This seemed to come natural to Jim, and was in display in the open and stakeholder-focused way he had set-up and was running the national MRC Biomedical NMR Centre, in the way he was running the Division he was leading, and in his generous contribution as an editor of the Progress in NMR Spectroscopy journal, among many. As a postdoc in that division, it was a privilege to witness how Jim, a very British scientist, would naturally understand and could support the diverse and international colleagues around him (including myself) by virtue of his humanity. Jim was always available to share his in-depth understanding of molecular phenomena, his practical experience of research and his bottomless repertoire of British jokes. We learned much.

## Sir John Skehel, the crick institute, London United Kingdom

I was at the NIMR from 1969 to 2006 so I met Jim when he arrived with Sir Arnold Burgen from Cambridge. I got to know Jim best when we were group leaders with the Director Dai Rees. Before that, Jim was the gentleman who had an almost apologetic way of asking questions at seminars that were always to the point but were sometimes crushingly embarrassing for the speaker. In the groups Jim was in charge of Molecular Structure as well as the NMR Centre, and the Collaborative Centre. His reports for Dai on the workings of the Collaborative Centre were meticulous. His science and administration of the NMR Centre shared this quality, the latter particularly characterized by his scrupulously fair division of time on the instruments between Institute and external scientists. His jealously guarded preparations for the reviews of the Centre were a model for the rest of the Institute and were largely responsible for its continued support and its survival to this day. Most of all Jim was a first-class scientist and a gentle man and for me personally and for the Institute, he was enormously supportive.

## Footnotes

1 J.W. Emsley and D. Neuhaus, Progress in NMR Spectroscopy, 150-151, 101584 (2025).

2 Annalisa Pastore and Stephan Grzesiek https://ismar.org/2025/10/09/james-feeney-1936-2025/James Feeney 1936–2025.

